# Early whole blood transcriptional responses to radiation-attenuated *Plasmodium falciparum* sporozoite vaccination in malaria naïve and malaria pre-exposed adult volunteers

**DOI:** 10.1186/s12936-021-03839-3

**Published:** 2021-07-09

**Authors:** Fergal J. Duffy, Ying Du, Jason Carnes, Judith E. Epstein, Stephen L. Hoffman, Salim Abdulla, Said Jongo, Maxmillian Mpina, Claudia Daubenberger, John D. Aitchison, Ken Stuart

**Affiliations:** 1 Center for Global Infectious Disease Research, Seattle Children’s Hospital, Seattle, WA USA; 2 Malaria Department, Naval Medical Research Center, Silver Spring, MD USA; 3 Department of Medical Parasitology and Infection Biology, Clinical Immunology Unit, Swiss Tropical and Public Health Institute, 4002 Basel, Switzerland; 4 University of Basel, Petersplatz 1, 4001 Basel, Switzerland; 5 Sanaria Inc., Rockville, MD USA; 6 Ifakara Health Institute, Bagamoyo, Tanzania

## Abstract

**Background:**

Vaccination with radiation-attenuated *Plasmodium falciparum* sporozoites is known to induce protective immunity. However, the mechanisms underlying this protection remain unclear. In this work, two recent radiation-attenuated sporozoite vaccination studies were used to identify potential transcriptional correlates of vaccination-induced protection.

**Methods:**

Longitudinal whole blood RNAseq transcriptome responses to immunization with radiation-attenuated *P. falciparum* sporozoites were analysed and compared across malaria-naïve adult participants (IMRAS) and malaria-experienced adult participants (BSPZV1). Parasite dose and method of delivery differed between trials, and immunization regimens were designed to achieve incomplete protective efficacy. Observed protective efficacy was 55% in IMRAS and 20% in BSPZV1. Study vaccine dosings were chosen to elicit both protected and non-protected subjects, so that protection-associated responses could be identified.

**Results:**

Analysis of comparable time points up to 1 week after the first vaccination revealed a shared cross-study transcriptional response programme, despite large differences in number and magnitude of differentially expressed genes between trials. A time-dependent regulatory programme of coherent blood transcriptional modular responses was observed, involving induction of inflammatory responses 1–3 days post-vaccination, with cell cycle responses apparent by day 7 in protected individuals from both trials. Additionally, strongly increased induction of inflammation and interferon-associated responses was seen in non-protected IMRAS participants. All individuals, except for non-protected BSPZV1 participants, showed robust upregulation of cell-cycle associated transcriptional responses post vaccination.

**Conclusions:**

In summary, despite stark differences between the two studies, including route of vaccination and status of malaria exposure, responses were identified that were associated with protection after PfRAS vaccination. These comprised a moderate early interferon response peaking 2 days post vaccination, followed by a later proliferative cell cycle response steadily increasing over the first 7 days post vaccination. Non-protection is associated with deviations from this model, observed in this study with over-induction of early interferon responses in IMRAS and failure to mount a cell cycle response in BSPZV1.

**Supplementary Information:**

The online version contains supplementary material available at 10.1186/s12936-021-03839-3.

## Background

Despite the existence of effective anti-parasitic drugs, malaria remains a critical global health problem, estimated at causing 409,000 deaths and 229 million cases in 2019. Some 94% of cases were in Africa, where almost all infections were caused by *Plasmodium falciparum* [1]. Currently, the most advanced malaria vaccine, RTS,S, exhibits 28–36% efficacy in infants and children observed over an average time period of 4 years [2]. A more effective malaria vaccine would be a valuable tool for curbing malaria, especially given the emergence of resistance to frontline artemisinin combination therapy and development of insecticide-resistant mosquito vectors [3, 4]. Repeated natural malaria infections can result in acquisition of semi-protective immunity with persistent low level parasitaemia and primarily asymptomatic cases [5]. Serious malaria-related complications and death occur primarily in infants and children, prior to the development of partially protective immune responses [6]. However, acquisition of sterilizing immunity targeting the pre-erythrocytic stage of the parasite, resulting from immunization with radiation-attenuated malaria sporozoites, has been experimentally demonstrated in animal models and in humans [7–10].

Malaria sporozoites develop in the mosquito and are injected into the skin during a female mosquito blood meal from where they make their way to the liver and infect hepatocytes. There they multiply and over the course of 5–9 days, asymptomatically develop into thousands of merozoites which emerge from the liver and serially infect erythrocytes, resulting in blood-stage infection and disease. The pre-erythrocytic stage initiated by sporozoites is a population bottleneck in the parasite lifecycle, and is an attractive target for vaccine development strategies. It was first demonstrated in a mouse model in 1967 that immunization with radiation-attenuated sporozoites (RAS) results in effective protective immunity against challenge with infectious sporozoites [10], and demonstrated subsequently for *P. falciparum* RAS (PfRAS) in human cohorts in multiple clinical trials [7, 11].

The immune mechanisms of human protection resulting from immunization with whole-sporozoite vaccines remain poorly understood but available evidence indicates that the development of this immunity requires liver infection. Work in animal models shows important roles associated with protection for antibodies, liver resident CD8+ memory T cells (Trm) and type I interferon responses [12–14]. However, results in animal models may not directly translate to humans, and the ability to directly monitor responses in human liver during vaccination and after controlled human malaria infection (CHMI) is very limited. Blood represents an accessible and immunologically important tissue which is reflective of systemic immune responses and its analysis can aid investigation of immune protection against malaria.

Two human RAS vaccination trials that resulted in a portion of the trial participants being protected from infection following CHMI have been performed, allowing comparisons between protected and non-protected subjects. Immunization by mosquito bite with radiation attenuated sporozoites (IMRAS) [15], [NCT01994525] and Bagamoyo sporozoite vaccine 1 study; immunizations with Sanaria^®^ PfSPZ Vaccine (BSPZV1) [16] trials, both included immunization of volunteers with five consecutive PfRAS vaccinations followed by homologous CHMI using *P. falciparum* strain NF54. Whole blood was sampled repeatedly from participants and analysed by RNAseq to provide longitudinal data on the immune responses. These trials differed with respect to malaria experience of the volunteers and route of administration for both immunization and CHMI, as described in “Methods”. This study analysed comparable time points in both trials up to 1 week after the first RAS vaccination. Shared transcriptional responses were identified in volunteers participating in both studies after initial RAS vaccination, indicating the existence of an initial core transcriptional response programme to RAS vaccination across diverse populations. Deviations from this transcriptional programme were associated with lack of protection.

## Methods

### Challenge trials

The IMRAS trial was performed in Bethesda, MD, USA with malaria-naive participants immunized by four rounds of ~ 190 bites of PfRAS-infected mosquitoes at 4-week intervals followed by a fifth immunization a further 5 weeks later. Protection was assessed 3 weeks after last vaccination by homologous CHMI via bites of five mosquitoes carrying non-irradiated *P. falciparum* sporozoites. This intentionally suboptimal immunization schedule resulted in 55% protection among the IMRAS cohort examined in this study.

The BSPZV1 trial was carried out in Bagamoyo, Tanzania with volunteers who potentially had prior malaria infections, but were free of symptomatic malaria for the previous 2 years. They were immunized 5 times by direct venous injection (DVI) with either 1.35 × 10^5^ or 2.7 × 10^5^ PfSPZ of Sanaria^®^ PfSPZ Vaccine (aseptic purified cryopreserved PfRAS) given at 4-week intervals. This was followed by CHMI at 3 weeks after the final immunization via DVI with PfSPZ Challenge (NF54) (purified cryopreserved non-irradiated SPZ), at a dose of 3200 PfSPZ. The resultant protective efficacy was 20%. Because not all non-protected individuals were included in RNAseq analysis, there was 36% protection among analysed individuals.

Total RNA was extracted from whole blood in PAXgene Blood RNA tubes that had been stored at – 80 °C, using the PAXgene Blood Kit (PreAnalytiX) following the manufacturer’s protocols. RNA was quantified by spectrophotometry and ~ 2.3 μg of total RNA per sample was processed using the GLOBINclear Human kit (Ambion) in order to remove globin mRNA. RNAseq was performed by Beijing Genomics Institute using either the Illumina Hiseq2000 (75 bp read length, paired-end), or the BGI500 platform (100 bp read length, paired-end) to a depth of at least 30 million reads per sample. Reads were aligned to the human hg19 genome using STAR [17], and htseq-count [18] using the intersection-strict option was used to convert mapped reads into a gene count table. Genes were filtered to retain only genes with > 10 read counts in at least 5% of samples. Counts were then normalized using the R limma [19] and voom [20] packages to account for sequencing depth and log2 transformed for all downstream analysis.

### Linear mixed-model analysis

The R lme4 [21] package was used to fit nested mixed models to assess differential gene expression in both cohorts, using the lmer function. Random intercepts were fit per-subject to account for multiple samples being drawn from the same study subjects. Five mixed models were fit to each gene, separately for differential expression at each sample day relative to the previous sample day in each study. For IMRAS these time intervals were days 1 vs 0, 3 vs 1, 7 vs 3, and for BSPZV1 days 2 vs 0 and 7 vs 2. Time and protection status were encoded as binary variables.

Formulae were fit as follows:

1 $$geneExpr\sim 1 + Time*Protection + \left( {1|Subject} \right)$$

2 $$geneExpr\sim 1 + Time + \left( {1|Subject} \right)$$

3 $$geneExpr\sim 1 + Protection + \left( {1|Subject} \right)$$

This model structure assumes that individual transcriptional responses are a function of (vaccine induced) responses over a time interval, and individual protection status. In the full model (1), both time and protection status are included. Reduced models comprising only time (2) or protection status (3) were also fit and, to assess the statistical significance of these parameters of interest, p-values associated with time- or protection-associated gene responses were determined by contrasting full models with reduced models. Specifically, p-values for time-associated genes were calculated by ANOVA to determine whether Eq. (1) significantly improved fit to the data compared to Eq. (3) using the anova.merMod() function. Similarly, Protection p-values were obtained by comparing Eq. (1) to Eq. (2). Response gene p-values were then false-discovery rate adjusted for multiple testing.

Directionality (UP, DN or NC (no change)) of each gene response was assessed using confidence intervals (CIs). The 90% CIs were estimated for the coefficient of Eq. (2), cases where the lower CI > 0 were considered UP genes, upper CI < 0 were considered DN genes.

Significant response genes were identified as those that met an false discovery rate (FDR) threshold < 0.2 a nominal p-value < 0.05, and were classified as UP or DN in direction. Candidate protection-associated genes were filtered to only include significant response genes, then false discovery rate adjusted. Protection genes were selected that met an FDR threshold < 0.33 and nominal p-value < 0.05. A more permissive FDR threshold was selected for response genes vs protection genes to reflect reduced statistical power comparing protection status within a timepoint vs comparing gene expression for all samples over a time interval.

### Gene-set enrichment analysis

Previously published coherent blood transcriptional modules (BTMs) published by Li et al. [22], Chaussabel et al. [23], and the MsigDB Hallmark collection [24] were used for gene set enrichment analysis (GSEA) [25] of whole blood RNAseq profiles. A total of 656 BTMs were tested. Samples were grouped by sample time interval (as above), study, and protection status. Genes were ranked by calculating the average change in normalized expression for each gene over each time interval. These rankings were used as input to calculate GSEA normalized enrichment scores (NES) with accompanying p-values, nominal and FDR adjusted. GSEA was performed using the R fgsea package [26], with 10,000 random permutations. GSEA NES that did not pass the significance cut-off FDR < 0.05 were set to 0 for all further analysis. Correlations between sub-group module NES profiles were assessed using Spearman’s rho.

### Spline curve fitting

Gene-averaged responses for each BTM were calculated using the 25% trimmed mean of BTM gene expression per sample. Responses were made relative to day 0 for each subject by subtracting the subject day 0 BTM expression from subsequent days. A spline curve was fit to all IMRAS and BSPZV1 samples at all time points for each BTM, using the R smooth.spline function with 3 degrees of freedom. The 99% CIs around each spline were calculated using 500 bootstrap replicates, taking approximately 2/3 of samples for each replicate. To calculate CI deviation from the 0 response line, lower and upper 99% CIs were assessed at each time point measured, i.e., days 1, 2, 3, and 7. The BTM response was considered significant if both lower and upper CIs were either above or below the 0 response line and the magnitude of the difference was defined as: if upCI < 0, upCI else lowCI.

## Results

### Study design

Participants were recruited as part of two independent RAS-vaccination studies: IMRAS, [NCT01994525], [15] and BSPZV1 [16]. Both trials comprised 5 immunizations with identical strains of *P. falciparum*, with protection assessed by homologous CHMI. In the case of BSPZV1, immunizations and CHMI were delivered intravenously as cryopreserved purified sporozoites while for IMRAS, irradiated sporozoites and CHMI were administered by mosquito bite. The IMRAS trial was performed in malaria-naive adults in the Bethesda, MD, USA, while BSZPV1 was conducted in Tanzanian adults with previous malaria experience. Longitudinal whole-blood RNAseq transcriptional profiles were obtained from the studies (Table 1). Comparable samples cross-study comprised 33 RAS-vaccinated participants, with 3 (BSPZV1) or 4 (IMRAS) time points per person, measured immediately prior to and up to 7 days after the initial RAS vaccination.

**Table 1:** Study composition

	IMRAS	BSPZV1
Study location	Bethesda, MD, USA	Bagamoyo, Tanzania
RAS delivery	Mosquito bite	Venous injection
Prev. malaria exposure	Naïve	Malaria free for > 2 years
N. protected	6	8
N. non-protected	5	14
RNAseq time-points		
D000	X	X
D001	X	
D002		X
D003	X	
D007	X	X

Table lists participants for whom longitudinal whole blood RNAseq profiles are available

### IMRAS and BSPZV1 share a small but significant overlap of vaccine-induced genes

Mixed-effects linear modelling was used to identify differentially expressed genes (DEGs) that significantly respond over any time interval after PfRAS vaccination. For IMRAS, these time intervals were day 0 (immediately prior to vaccination) to day 1 (post vaccination), day 1 to day 3, and day 3 to day 7. For BSPZV1, time intervals were day 0 to day 2, and day 2 to day 7. Significant DEGs (FDR < 0.2) over a time interval were identified using a nested mixed modelling approach (see “Methods”) and classified as increased or decreased based on 90% CIs of the model time coefficient. This accounted for dynamic expression changes between subsequent sampling times. Subsequently, the changes in the DEGs over time intervals were tested for significant associations with protection (FDR < 0.33).

**Fig. 1:**
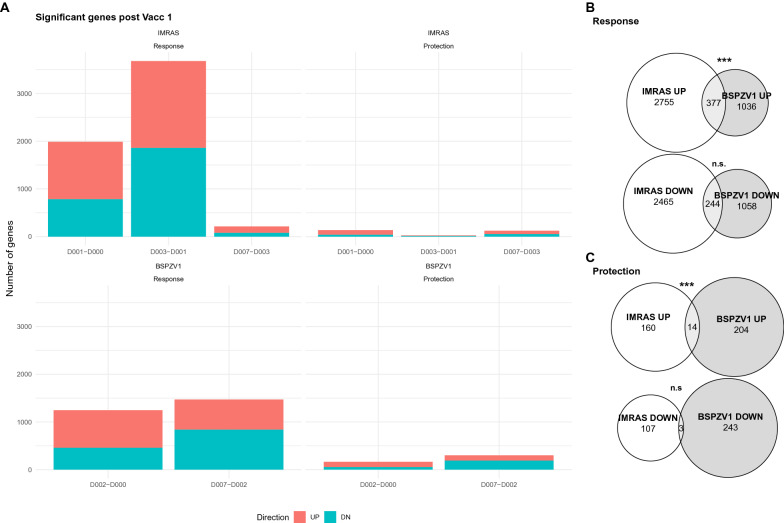
Limited cross-study overlap of differentially expressed genes. **A** Counts of significantly up- and down-regulated genes for each time interval up to 1 week after first RAS vaccination for both IMRAS and BSPZV1, including both vaccine response and protection-associated genes. **B**, **C** Venn diagrams showing intersection of up and down-regulated response (**B**) and protection (**C**) associated genes between IMRAS and BSPZV1. Annotations indicate hypergeometric test p-value of gene overlap: ‘***’ p < 0.001; ‘n.s.’ p > 0.05

The numbers of genes that significantly increased or decreased at each time interval for each study are shown in Fig. 1a. Overall, IMRAS showed 2–3× more time-interval associated DEGs (UP: 3133, DN: 2709) compared with BSPZV1 (UP: 1413, DN: 1302) (Fig. 1b). However, a larger proportion of BSPZV1 time-interval DEGs differed in expression between protected and non-protected subjects (UP: 174, DN: 218) compared with IMRAS (UP:174, DN:110) (Fig. 1a). A modest but significant overlap was observed (377 genes) in UP, but not DN responses and genes between the trials (p = 2.02 × 10^–4^, Table 2, Fig. 1c). Fourteen of these genes were associated with protection, also representing a significant increase in what would be expected to be shared by random overlap in the gene lists (p = 7.24 × 10^–7^, Table 2, Additional file 1: Table S1). While this overlap represented a small minority of genes responding in each study, it pointed to conserved upregulated responses early after PfRAS vaccination.

**Table 2:** Limited cross-study overlap of differentially expressed genes

			N shared genes	N expected	p
Response	IMRAS UP	BSPZV1 UP	377	323	2.02 × 10^–4^
Response	IMRAS DOWN	BSPZV1 DOWN	244	257	0.84
Protection	IMRAS UP	BSPZV1 UP	14	3	7.24 × 10^–7^
Protection	IMRAS DOWN	BSPZV1 DOWN	3	2	0.32

Hypergeometric test p-values and overlap sizes for the up- and down-regulated genes identified by mixed modelling

In order to identify more broadly shared transcriptional response pathways between studies, previously published sets of transcriptionally coherent BTMs [22–24] were used. The 377 shared cross-study DEGs (UP in both IMRAS and BSPZV1) were assessed for BTM enrichment using the hypergeometric test. 35 significantly enriched BTMs (FDR < 0.1, Table 3) were identified, from a variety of functional classes, including erythrocytes, cell cycle and inflammatory modules. Six of these BTMs were also specifically enriched in the 14 upregulated cross-study protection associated genes (Table 3). Notably, 8 of 14 protection-associated genes, SPAG5, EZH2, NCAPH, HJURP, NUSAP1, DTL, CKAP2L, and RRM2 were members of a single BTM: LI.M4.0_cell cycle and transcription (Additional file 1: Table S1). A further two, HAGH and CARM1, were part of the DC.M3.1_Erythrocytes BTM. Several other protection-associated genes were part of immune-related BTMs: AGPAT3 in LI.S5_DC surface signature, LGALS3BP in DC.M3.4_Interferon, CHKA in DC.M7.1_Inflammation. CREBL2 was the sole protection-associated gene not found in a well-annotated BTM; it was only found in the DC.M8.8_Undetermined BTM.

**Table 3:** Blood transcriptional modules enriched in overlapping differentially expressed genes

N genes	N BTM	N shared	N expected	p value	FDR	Blood transcriptional module (BTM) name	Protection associated
377	82	26	2	4.58E−21	3.01E−18	DC.M3.1_Erythrocytes	
377	145	32	4	2.55E−20	8.35E−18	LI.M4.1_cell cycle (I)	*
377	335	44	9	3.53E−18	7.72E−16	LI.M4.0_cell cycle and transcription	*
377	49	17	1	6.00E−15	9.84E−13	DC.M3.3_Cell Cycle	*
377	77	20	2	1.31E−14	1.71E−12	DC.M4.4_Undetermined	
377	34	14	1	9.42E−14	1.03E−11	LI.M4.2_PLK1 signaling events	*
377	200	29	6	1.89E−13	1.77E−11	HALLMARK_E2F_TARGETS	*
377	200	27	6	7.41E−12	6.08E−10	HALLMARK_HEME_METABOLISM	
377	71	16	2	6.30E−11	4.59E−09	DC.M2.3_Erythrocytes	
377	35	11	1	1.36E−09	8.91E−08	LI.M4.5_mitotic cell cycle in stimulated CD4 T cells	
377	200	23	6	6.65E−09	3.97E−07	HALLMARK_G2M_CHECKPOINT	
377	51	10	1	1.03E−06	5.61E−05	LI.M103_cell cycle (III)	
377	32	8	1	1.79E−06	9.05E−05	LI.M6_mitotic cell division	
377	47	9	1	4.42E−06	2.07E−04	LI.M49_transcription regulation in cell development	
377	12	5	0	1.03E−05	4.53E−04	LI.M4.12_C-MYC transcriptional network	
377	20	6	1	1.16E−05	4.77E−04	DC.M6.11_Cell Cycle	
377	21	6	1	1.59E−05	6.13E−04	LI.M4.7_mitotic cell cycle	
377	14	5	0	2.50E−05	9.11E−04	LI.M4.10_cell cycle (II)	*
377	97	11	3	7.04E−05	2.43E−03	DC.M5.3_Undetermined	
377	245	18	7	1.51E−04	4.97E−03	DC.M5.5_Undetermined	
377	59	8	2	1.98E−04	6.18E−03	DC.M4.14_Monocytes	
377	34	6	1	2.91E−04	8.68E−03	DC.M7.31_Undetermined	
377	14	4	0	4.53E−04	1.29E−02	LI.M173_erythrocyte differentiation	
377	26	5	1	6.26E−04	1.71E−02	DC.M8.26_Undetermined	
377	16	4	0	7.88E−04	2.07E−02	LI.M4.9_mitotic cell cycle in stimulated CD4 T cells	*
377	17	4	0	1.01E−03	2.45E−02	LI.M19_T cell differentiation (Th2)	
377	17	4	0	1.01E−03	2.45E−02	LI.M136_TBA	
377	33	5	1	1.93E−03	4.52E−02	LI.M10.0_E2F1 targets (Q3)	
377	10	3	0	2.14E−03	4.85E−02	LI.M4.14_Rho GTPase cycle	
377	11	3	0	2.89E−03	5.74E−02	LI.M4.15_enriched in monocytes (I)	
377	11	3	0	2.89E−03	5.74E−02	LI.M12_CD28 costimulation	
377	11	3	0	2.89E−03	5.74E−02	LI.M33_inflammatory response	
377	11	3	0	2.89E−03	5.74E−02	LI.M171_heme biosynthesis (I)	
377	12	3	0	3.77E−03	7.28E−02	LI.M4.11_mitotic cell cycle in stimulated CD4 T cells	
377	13	3	0	4.81E−03	9.01E−02	LI.M15_Ran mediated mitosis	

Hypergeometric p-values and overlap sizes for blood transcriptional modules enriched in shared IMRAS and BSPZV1 upregulated DEGs. BTMs enriched in the 14-gene protection associated subset are indicated by as asterisk (*)

### Gene-set enrichment analysis reveals shared response pathways between studies

Module enrichment based on DEGs was limited by the statistical power to accurately identify differentially expressed genes, and did not take into account responses specific to either IMRAS or BSPZV1. To expand on the previous analysis and more broadly identify transcriptional response pathways, BTM responses were assessed using GSEA separately for BSPZV1 and IMRAS, and for protected: P and non-protected: NP individuals. In other words, GSEA was performed on sample sub-groups: one sub-group per study (BSPZV1 or IMRAS) per protection status per time interval (defined as for the DEG analysis), for a total of 10 sub-groups. This level of stratification was chosen to reveal all potential combinations of study- and protection-specific BTM responses. In contrast to DEG-based enrichment analysis, GSEA takes into account the rank expression level of all detectable genes in the transcriptome.

**Fig. 2:**
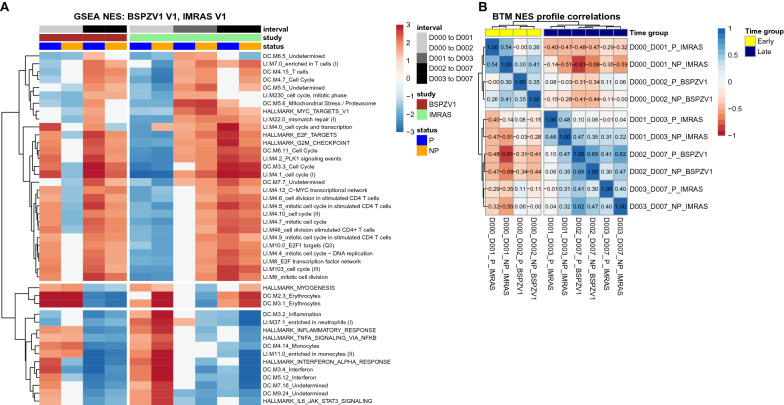
GSEA reveals shared co-ordinated cross-study responses up to 1 week after 1st RAS vaccination. **A** Heatmap shows GSEA normalized enrichment scores (NES) for modules significantly enriched across at least 8 of 10 total time interval/protection groups. **B** Hierarchically clustered correlation matrix indicating Spearman correlations between GSEA NES scores for each time interval/protection group. Black rectangles indicate two largest hierarchical subclusters, corresponding to early (day 1 or 2) and late (day 3 or 7) responses

GSEA NES were calculated separately for each sub-group. Figure 2a shows GSEA NES for significant BTMs (FDR < 0.05) in at least 8 of the 10 total sub-groups. Hierarchical clustering revealed related BTM responses clustered closely together, showing similar time and protection-associated responses for functionally similar modules. Three common cross-study response groupings were apparent from hierarchical clustering, representing inflammatory/interferon responses: erythrocytes/myogenesis; cell cycle responses. Interferon-associated BTM responses were increased in the day 0–1 (IMRAS) or day 0–2 (BSPZV1) time intervals after vaccination, while cell cycle responses were most increased in the day 2/3–7 time intervals. Protection-associated and trial-cohort specific differences were also apparent, with IMRAS NP subjects showing increased interferon responses relative to P in the day 0–1 interval, while these differences were not observed in BSPZV1 participants. In addition, BSPZV1 P subjects exhibited upregulated cell cycle responses in the day 0–2 interval that was not evident in BSPZV1 NP subjects. Induction of cell-cycle responses in IMRAS over the day 0–1 interval was not observed, and cell cycle responses between days 1 and 3 in IMRAS were primarily apparent in NP individuals.

Figure 2b shows correlations between BTM NES values per sub-group. The sub-groups have been arranged by hierarchical clustering, revealing two positively correlated cross-study response sub-groups, consisting of ‘early’ responses in the day 1 or 2 intervals post vaccination for P and NP subjects in both BSPZV1 and IMRAS; and ‘late’ responses in the day 7 intervals post vaccination. Importantly, response profiles from both IMRAS and BSPZV1, and from P and NP subjects clustered together by time, suggesting a shared temporal response program after RAS vaccination for both IMRAS and BSPZV1.

### Temporal modelling reveals time dynamics of cross study BTM responses

**Fig. 3:**
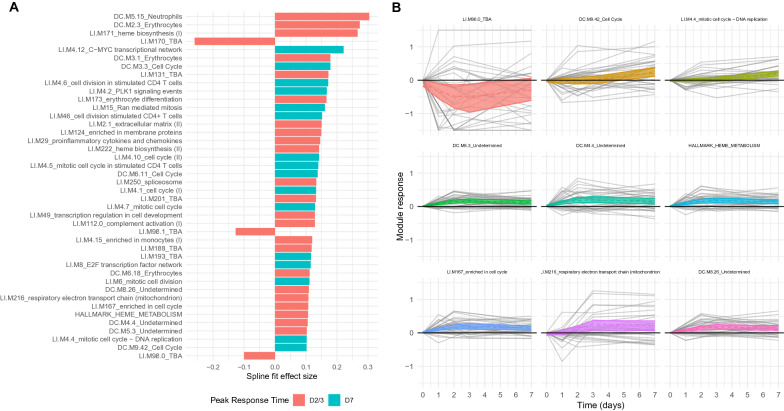
Temporal modelling reveals time dynamics of cross study module response. **A** Bar plot of module response effect sizes, i.e. maximum separation of spline fit 99% CI range from average day 0 level. Colors indicate sample day closest to maximum module response. All modules with absolute effect sizes > 0.1 shown. **B** Top 9 module response spline fits (dashed line) with 99% CIs (ribbon). Individual subject module responses from IMRAS and BSPZV1 are shown as light grey lines

Given the observed overlap in gene and BTM responses between IMRAS and BSPZV1, it was hypothesized that a shared transcriptional response may be elicited by PfRAS in both trials in the days after PfRAS vaccination. However, the differing timepoints measured in IMRAS and BSPZV1 complicated direct day-by-day comparisons. Therefore, to directly explore time dynamics underlying cross-study BTM responses in IMRAS and BSPZV1, continuous spline curves were fit to averaged BTM responses. Samples from P and NP subjects from both IMRAS and BSPZV1 were combined, and BTM responses were calculated as the average of BTM gene expression, relative to the day of vaccination. The 99% CIs were calculated, and deviation of the CI from the zero-response line was used to identify significantly responsive BTMs. For significant BTMs, the time associated with the maximum response was used to classify modules into those whose response peaks at day 7 or those with a peak response reached by day 3. 42 BTMs showed significant responses (Fig. 3a), with the 9 most strongly changed BTMs also shown in Fig. 3b of which 39 increased and 3 decreased in expression post vaccination. The majority of BTMs reached their maximum response by 2 or 3 days post vaccination, with the exception being BTMs associated with cell-cycle processes, such as ‘LI.M4.12_C-MYC transcriptional network’ and ‘DC.M3.3_Cell Cycle.’

Overall, there was very strong overlap between BTMs which were identified as significantly responsive by three orthogonal approaches, i.e., enrichment in DEGs, GSEA and temporal modelling. Additional file 2: Fig. S1 shows a proportional Venn diagram indicating the overlap in BTMs identified by each approach. For each of the three approaches: Mixed Model DEG enrichment, GSEA, and Curve fitting, at least half the modules revealed by any one approach were also identified by one or more alternate approaches, e.g. of the 44 BTMs identified by GSEA, 22 were also identified by DEG enrichment or curve fitting approaches, or both. A core set of 11 BTMs were identified by all three approaches (Additional file 2: Fig. S1), principally consisting of cell cycle related BTMs, one of which was specifically associated with mitotic cell cycle in stimulated CD4 T cells.

### Study specific responses correlate with protection and include baseline expression differences

**Fig. 4:**
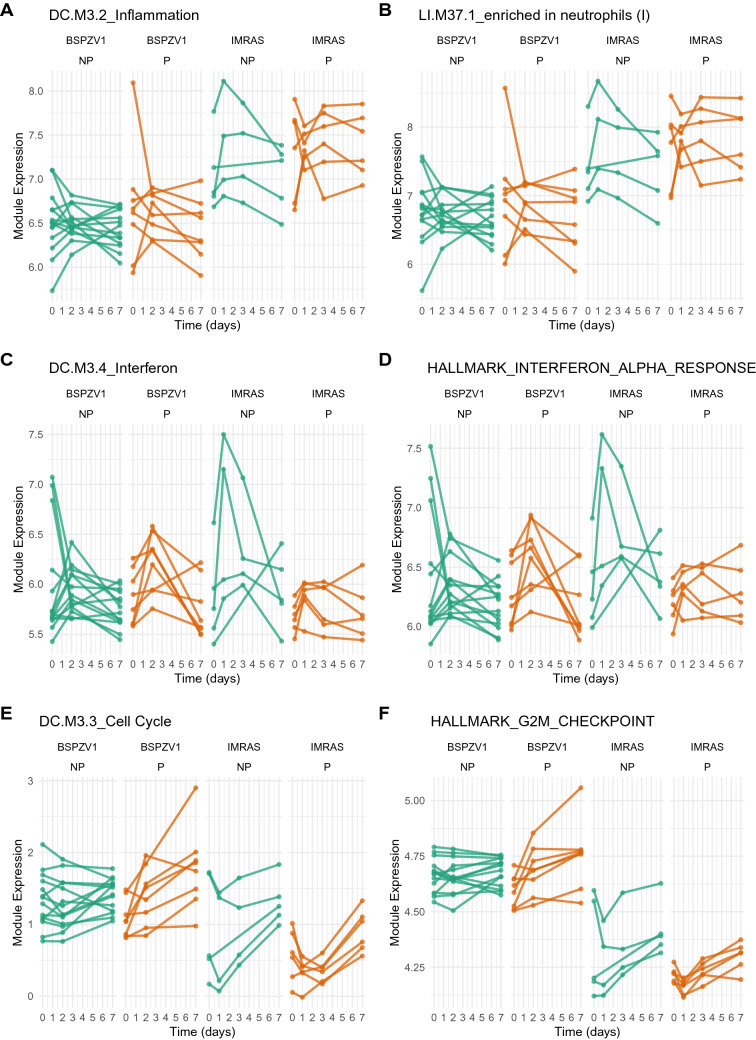
Protection associated responses differ between studies after vaccination and at baseline. **A**–**F** Average BTM responses for modules differentially expressed between studies at day 0 (**A**, **B**), associated with protection in IMRAS (**C**, **D**) or BSPZV1 (**E**, **F**)

GSEA analysis (Fig. 2) revealed gene sets responsive to vaccination in both studies and study-specific protection-associated differences between IMRAS and BSPZV1. Interferon and inflammatory responses were increased in IMRAS NP vs IMRAS P in the day 0 to 1 interval, while cell cycle responses were stronger in BSPZV1 P compared with BSPZV1 NP in the day 2 to 7 interval. Since both the shared DEG and curve fitting analysis were aimed at identifying shared cross-study responses, they could not have detected any study-specific differences. Six interferon, inflammatory and cell-cycle modules were selected from Fig. 2, and the average BTM expression was calculated (Fig. 4), showing distinct study-specific patterns. BTMs associated with inflammation and neutrophil signalling (Fig. 4a, b), were consistently more highly expressed in IMRAS compared with BSPZV1 over the entire study period. In contrast, interferon response BTMs (Fig. 4c, d) did not show stark differences at day 0; however, responses were specifically increased in IMRAS NP vs IMRAS P and BSPZV1 P and NP subjects. For cell-cycle associated modules (Fig. 4e, f), both BSPZV1 P and NP subjects showed higher expression at baseline relative to IMRAS; however, BSPZV1 NP individuals, uniquely, did not exhibit further increased cell cycle responses at any point after vaccination.

**Fig. 5:**
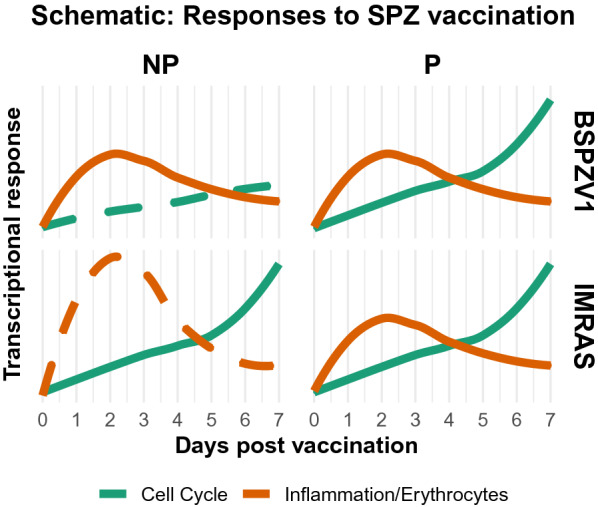
Proposed model for cross-study RAS responses. Line plots illustrate responses common to protected individuals in IMRAS and BSPZV1, along with study-specific deviations from these responses in non-protected individuals

Overall, synthesizing information from each approach suggests a model for responses consistent with protection after PfRAS vaccination, incorporating a moderate early interferon response peaking 2 days post vaccination followed by a later proliferative cell cycle response steadily increasing over the first 7 days post vaccination (Fig. 5). Non-protection is associated with deviations from this model, observed in this study with over-induction of early interferon responses in IMRAS and failure to mount a cell cycle response in BSPZV1.

## Discussion

Human malaria correlates of protection/response to PfRAS vaccination have been difficult to identify. This may be due to the mechanisms of PfRAS-mediated protection because PfRAS vaccination results in a truncated liver stage parasite development, with no subsequent blood stage. Presumably, key protective immune processes happen at the site of infection, in the liver, where direct measures of responses are limited. This study represents the first time, to the authors’ knowledge, that common transcriptional correlates of RAS vaccination have been identified in multiple cohorts. This was done via analysis of whole blood RNAseq after vaccination, which reflects systemic immune responses.

The time-points examined here, up to 1 week post vaccination, were too early to capture the adaptive immune response to vaccination. Therefore, it is likely that the shared response identified here represents an effective innate immune response capable of presenting antigen and kickstarting the adaptive response. Whole blood RNAseq can detect systemic responses to PfRAS vaccination but is limited by the fact that sporozoite antigen specific cells represent a small minority of circulating blood cells. Additionally, whole blood RNAseq reflects the transcriptional state of every circulating leukocyte, and it is not possible to definitively link changes in expression to immune cell populations. Neither can it be determined whether changes in transcription reflect expansions or decreases in numbers of immune cells, or changes in transcriptional state of specific cell types. Despite these limitations, modules identified suggest an initial innate immune response followed by upregulation of cell cycle BTMs which may reflect rapid innate detection of PfRAS, followed by immune cell activation and proliferation. That the majority of the 14 shared protection associated genes identified were also associated with innate immunity or cell cycle suggests that deviations from the coordinated cross-study response programme are associated with non-protection. Recent work by Tran et al*.* [27] on whole sporozoite vaccination by administration of infectious sporozoites under chloroquine prophylaxis observed changes in T-cell associated BTMs in protected individuals 3 weeks after initial vaccination. This suggests that an extended series of cross-study comparable time-points may reveal adaptive immune associated responses. Unlike this study, they did not see significant changes in BTM expression within 1 week, however their study design did not include any sampling within 1–5 days post vaccination. They did observe increase of inflammatory/interferon and DC activation processes specifically in non-protected individuals 9 days after the third CPS immunization. Despite the difference in timescale, this shares some similarity with the IMRAS study, where over-induction of interferon is associated with non-protection. Recent work in a mouse model also supports the hypothesis that excessive type-I interferon inhibits the production of malaria-specific IFNγ producing CD8+ T cells [13]. In contrast, transcriptional profiling of RTS,S vaccinees revealed multiple BTMs correlated with immunogenicity and protection within 1 week of vaccination, including cell cycle and inflammatory response BTMs [28]. In contrast to the results seen after whole-sporozoite immunization, expression of interferon-associated and inflammatory-associated genes was positively associated with protection after RTS,S vaccination, and this is especially evident during the week after the second immunization. This may reflect differing pathways to protection induced by whole-sporozoite and subunit vaccines.

Stark differences in protective efficacy were observed between IMRAS, where 55% of study subjects were protected after intentional suboptimal immunization, and BSPZV1 where only 20% of the subjects were protected from CHMI. Although 20% of BSPZV1 subjects overall were protected, the RNAseq analysis was performed on a subset of BSPZV1 participants in which 36% were protected (Table 1). Unlike vaccination with PfRAS, it has long been observed that repeated natural malaria infection does not lead to sterilizing immunity [29–34]. Reasons for this are unclear; however, it may be associated with low doses of sporozoites delivered in natural infections or active immune evasion strategies mounted by the parasite. Indeed, it has been observed that blood stage malaria inhibits or actively dysregulates the development of effective CD8+ T-cell and antibody-mediated liver stage immunity [35, 36]. Previous immune exposure may be a factor in the observed reduction in protective efficacy of PfRAS vaccination in BSPZV1 vs IMRAS. Thus, it is plausible that the functional pathways leading to antigen presentation and adaptive immune priming may operate using different mechanisms in both trials. In other words, the different delivery method, dose, and previous malaria experience of BSPZV1 participants may have led to a qualitatively different, or non-naive, immune response to PfRAS in BSPZV1 compared to that observed in IMRAS. Therefore, true correlates of protection may exist that are distinct to both studies and would not be captured by this joint analysis.

Host intrinsic factors, e.g., genetic differences, and extrinsic factors, e.g., co-morbidities, microbiome, and general immune status, could also have contributed to the differences between IMRAS and BSPZV1. However, reduced levels of PfRAS-induced protection have been previously observed in malaria-experienced vs malaria-naive adults [9] suggesting that this issue is not specific to these two study cohorts. Intriguingly, IMRAS showed increased expression of inflammation associated BTMs pre-immunization, compared with BSPZV1. This may influence the induction of inflammatory responses post RAS vaccination, and a more effective initial innate response may partially explain better protection in IMRAS.

However, other differences between the two trials are likely to be associated with protection status. IMRAS participants received PfRAS vaccination and PfSPZ infection via mosquito bite, while BSPZV1 used cryopreserved parasites administered intravenously. A comparison of protection vs non-protection does not capture any potential immune response to mosquito bites, independent from PfRAS, in the IMRAS cohort. Previous work suggest that mosquito saliva can affect T cell and NK cell populations potentially up to 7 days post-bite [37]. The doses of PfRAS are very difficult to compare between trials, as IMRAS PfRAS doses were measured in terms of numbers of bites from infected irradiated mosquitos (~ 200 per vaccination), while BSPZV1 injected precise numbers of cryopreserved PfRAS. Another potential consequence of cryopreservation may be reduced PfRAS viability. Additionally, cryopreserved parasites were injected directly into the circulation while mosquito bites deliver PfRAS into the skin. Altogether, these differences would have resulted in differences in the number of hepatocytes that were infected by PfRAS during vaccination, and the number of PfRAS cleared by the innate immune system without reaching the liver.

While this study revealed common and distinct differences in whole blood gene expression patterns that correlate with protection and non-protection between these two studies, there are limitations to this analysis. This work relied on two study cohorts, with small numbers of study participants, comprising a total of 33 individuals to draw our conclusions. In addition, this analysis is limited to systemic responses the time period shortly following prime RAS vaccination. While consistent cross-study responses were identified, differing mechanisms of non-protection in each study were observed that would ideally be validated by further RAS cohorts. To validate these observations, it would be expected that malaria-naive RAS cohorts behave similarly to IMRAS and malaria experienced cohorts behave similarly to BSPZV1.

## Conclusion

This work has produced a conceptual model of an innate immune response programme consistent with PfRAS-induced protection, based on cross-study responses in two diverse cohorts. These responses are evident early after PfRAS primary vaccination and may serve as correlates of efficacy for future attenuated sporozoite vaccine candidates. Future work will comprise characterization of cell phenotypic changes over the course of vaccination, identifying the cell types responsible for the transcriptional changes seen here and explore adaptive immune responses to identify antibody and T cell responses that mediate sterilizing immunity.

## Supplementary Information

**Additional file 1: Table S1.** Shared protection genes in IMRAS and BSPZV1, and associated BTMs.

**Additional file 2: Figure S1.** Intersection of modular approaches reveals core responsive modules. Proportional venn diagram shows the overlap of significant response modules identified by spline-curve fitting, GSEA, and hypergeometric tests for BTM enrichment in individual response genes identified by mixed modelling.

## Data Availability

All sequencing data analysed in this paper will be made available through the ImmPort portal (immport.org).

## Abbreviations

RAS: Radiation-attenuated sporozoites
PfRAS: *Plasmodium falciparum* Radiation attenuated sporozoites
CHMI: Controlled human malaria infection
BTM: Blood transcriptional module
P: Protected
NP: Non-protected
DEG: Differentially expressed gene
NES: Normalized enrichment score
GSEA: Gene-set enrichment analysis
CI: Confidence interval
DVI: Direct venous injection
IMRAS: IMmunization by mosquito bite with Radiation Attenuated Sporozoites study
BSPZVI: Bagamoyo Sporozoite Vaccine 1 Study
DEG: Differentially Expressed Gene
UP: Up
DN: Down
NC: No change
