# Supplementary material for: Early whole blood transcriptional responses to radiation-attenuated Plasmodium falciparum sporozoite vaccination in malaria naïve and malaria pre-exposed adult volunteers
Source: Malar J. 2021 Jul 9;20:308. doi: 10.1186/s12936-021-03839-3 (PMC8267772; doi:10.1186/s12936-021-03839-3)
Supplement: Supplementary file 2 — Additional file 2: Figure S1. Intersection of modular approaches reveals core responsive modules. Proportional venn diagram shows the overlap of significant response modules identified by spline-curve fitting, GSEA, and hypergeometric tests for BTM enrichment in individual response genes identified by mixed modelling. [file 12936_2021_3839_MOESM2_ESM.pdf]

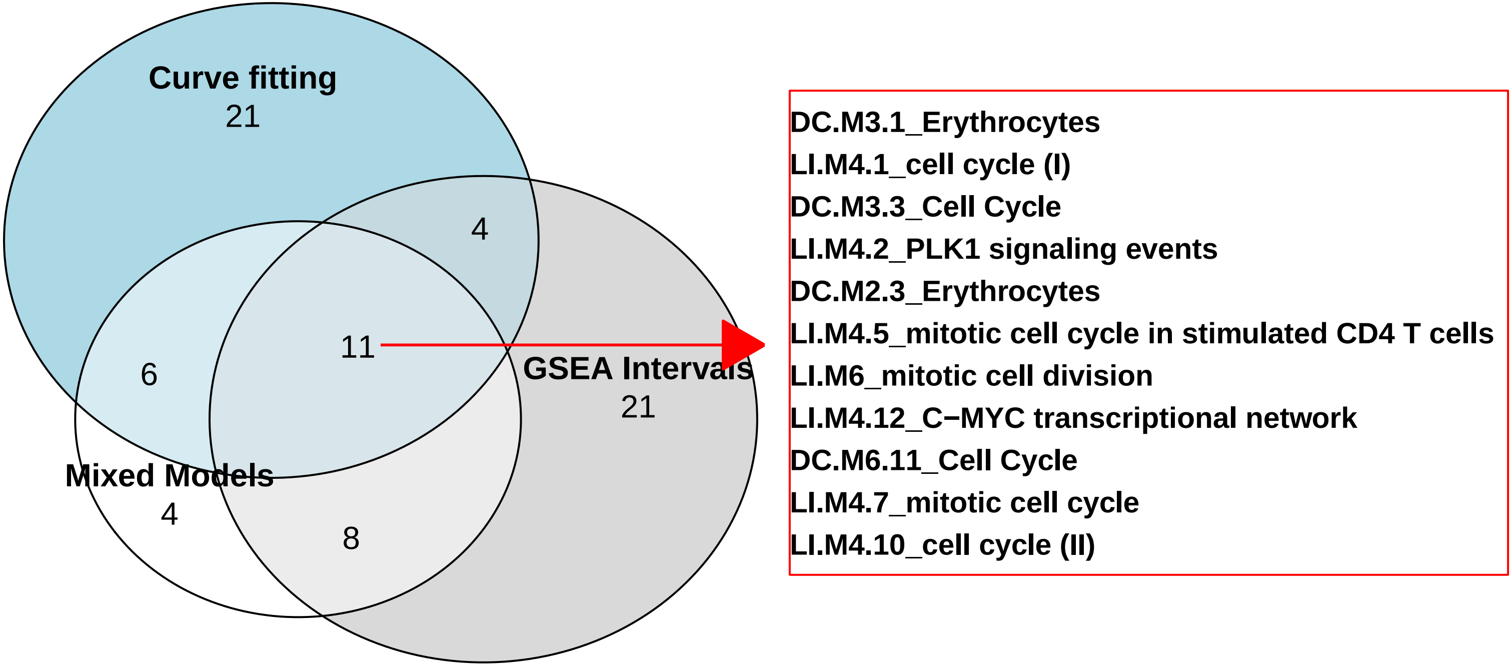
Curve fitting
21
4
11
6
GSEA Intervals
21
Mixed Models
4
8
DC.M3.1_Erythrocytes
LI.M4.1_cell cycle (I)
DC.M3.3_Cell Cycle
LI.M4.2_PLK1 signaling events
DC.M2.3_Erythrocytes
LI.M4.5_mitotic cell cycle in stimulated CD4 T cells
LI.M6_mitotic cell division
LI.M4.12_C−MYC transcriptional network
DC.M6.11_Cell Cycle
LI.M4.7_mitotic cell cycle
LI.M4.10_cell cycle (II)
